# Supplementary material for: End Sequence Analysis Toolkit (ESAT) expands the extractable information from single-cell RNA-seq data
Source: Genome Res. 2016 Oct;26(10):1397–410. doi: 10.1101/gr.207902.116 (PMC5052061; doi:10.1101/gr.207902.116)
Supplement: Supplemental Material [file supp_gr.207902.116_Supp_Materials.tar.gz › ESAT-esat_v0.1/doc/html/classbroad_1_1pda_1_1feature_1_1genome_1_1_chromosome-members.html]

ESAT: Member List


ESAT


- Main Page
- Related Pages
- Packages
- Classes
- Files

- Class List
- Class Index
- Class Hierarchy
- Class Members

All Classes Namespaces Files Functions Variables Enumerator Pages

- broad
- pda
- feature
- genome
- Chromosome

broad.pda.feature.genome.Chromosome Member List

This is the complete list of members for broad.pda.feature.genome.Chromosome, including all inherited members.

|  |  |  |
| --- | --- | --- |
| Chromosome(final String name) | broad.pda.feature.genome.Chromosome |  |
| Chromosome(String name, String agpFile) | broad.pda.feature.genome.Chromosome |  |
| compareNames(String name1, String name2) | broad.pda.feature.genome.Chromosome | static |
| compareTo(Object o) | broad.pda.feature.genome.Chromosome |  |
| delete(SequenceRegion region) | broad.pda.feature.genome.Chromosome |  |
| drawRandomRegion(int size) | broad.pda.feature.genome.Chromosome |  |
| extractRegion(SequenceRegion region) | broad.pda.feature.genome.Chromosome |  |
| extractRegions(List<?extends SequenceRegion > regions) | broad.pda.feature.genome.Chromosome |  |
| gaps() | broad.pda.feature.genome.Chromosome |  |
| getChromosomeNumber() | broad.pda.feature.genome.Chromosome |  |
| getChromosomeNumber(String name) | broad.pda.feature.genome.Chromosome | static |
| getName() | broad.pda.feature.genome.Chromosome |  |
| getRegion(SequenceRegion region) | broad.pda.feature.genome.Chromosome |  |
| getRegion(SequenceRegion region, boolean softmask) | broad.pda.feature.genome.Chromosome |  |
| getRegion(SequenceRegion region, boolean softmask, Map< String, IntervalTree< Alignments >> okRepeats) | broad.pda.feature.genome.Chromosome |  |
| getRegions(List<?extends GenomicAnnotation > annotations, int padding) | broad.pda.feature.genome.Chromosome |  |
| getSequence() | broad.pda.feature.genome.Chromosome |  |
| getShortArm() | broad.pda.feature.genome.Chromosome |  |
| getSymbol() | broad.pda.feature.genome.Chromosome |  |
| getUngappedSize() | broad.pda.feature.genome.Chromosome |  |
| insert(SequenceRegion region, LightweightGenomicAnnotation insertPoint) | broad.pda.feature.genome.Chromosome |  |
| invert(SequenceRegion region) | broad.pda.feature.genome.Chromosome |  |
| isAutosome() | broad.pda.feature.genome.Chromosome |  |
| isAutosome(String name) | broad.pda.feature.genome.Chromosome | static |
| isSexChromosome() | broad.pda.feature.genome.Chromosome |  |
| isSexChromosome(String name) | broad.pda.feature.genome.Chromosome | static |
| length() | broad.pda.feature.genome.Chromosome |  |
| loadSequence() | broad.pda.feature.genome.Chromosome |  |
| setSequenceFile(File file) | broad.pda.feature.genome.Chromosome |  |
| shuffle(GenomicAnnotation toShuffle, List<?extends GenomicAnnotation > toAvoid) | broad.pda.feature.genome.Chromosome |  |
| shuffle(List<?extends GenomicAnnotation > toShuffle, List<?extends GenomicAnnotation > toAvoid) | broad.pda.feature.genome.Chromosome |  |
| shuffleWithinValid(Annotation toShuffle, List<?extends Annotation > valid) | broad.pda.feature.genome.Chromosome |  |
| shuffleWithinValid(List<?extends Annotation > toShuffle, List<?extends Annotation > valid) | broad.pda.feature.genome.Chromosome |  |
| unloadSequence() | broad.pda.feature.genome.Chromosome |  |


---

Generated on Fri Aug 15 2014 15:10:59 for ESAT by  

 1.8.7
